# Direct-Drive Electro-Hydraulic Servo Valve Performance Characteristics Prediction Based on Big Data and Neural Networks

**DOI:** 10.3390/s23167211

**Published:** 2023-08-16

**Authors:** Juncheng Mi, Jin Yu, Guoqin Huang

**Affiliations:** College of Mechanical and Vehicle Engineering, Chongqing University, Chongqing 400044, China; 20142484@cqu.edu.cn (J.M.); huangguoqin@cqu.edu.cn (G.H.)

**Keywords:** direct-drive electrohydraulic servo valves, performance degradation, erosive wear, machine learning, neural networks

## Abstract

Direct-drive electro-hydraulic servo valves play a key role in aerospace control systems, and their operational stability and safety reliability are crucial to the safety, stability, and efficiency of the entire control system. Based on the prediction of the performance change of the servo valve and the resulting judgement and prediction of its life, this can effectively avoid serious accidents and economic losses caused by failure due to performance degradation in the work. On the basis of existing research, factors such as opening, oil contamination, and pressure difference are used as prerequisites for the operation of direct-drive electro-hydraulic servo valves. In addition to the current research on pressure gain and leakage, the performance parameters of servo valves, such as overlap, threshold, and symmetry, are also expanded and selected as research objects, combined with pressure design servo valve performance degradation experiments for testing instruments such as flow and position sensors, and data are obtained on changes in various performance parameters. The experimental data are analyzed and a prediction model is built to predict the performance parameters of the servo valve by combining the existing popular neural networks, and the prediction error is calculated to verify the accuracy and validity of the model. The experimental results indicate that as the working time progresses, the degree of erosion and wear on the valve core and valve sleeve of the servo valve increases. Overall, it has been observed that the performance parameters of the servo valve show a slow trend of change under different working conditions, and the rate of change is generally higher under high pollution (level 9) conditions than under other conditions. The prediction results indicate that the predicted values of various performance parameters of the servo valve by the prediction model are lower than 0.2% compared to the experimental test set data. By comparing the two dimensions of the accuracy and prediction trend, this model meets industrial needs and outperforms deep learning algorithm models such as the exponential smoothing algorithm and ARIMA model. The experiments and results of this study provide theoretical support for the life prediction model of servo valves based on neural networks and machine learning in artificial intelligence, and provide a reference for the development of direct-drive electro-hydraulic servo valves in aerospace and other industrial fields for use and failure standards.

## 1. Introduction

With the rapid development of global science and technology, modern electromechanical systems are widely used in aerospace, military equipment, and other high-tech fields. Because of the special requirements of aerospace and military fields, high-tech electromechanical systems, as the core of technology, must have extremely high reliability and a long service life [1]. However, modern high-tech electromechanical systems involve multiple fields of science and technology, such as mechanical, electrical, hydraulic, and control disciplines, which are closely coupled with each other. During the operation of high-tech electromechanical systems, electromechanical systems’ composition and special working conditions may cause serious accidents, resulting in casualties and economic losses. Therefore, predicting the service life of high-tech electromechanical systems and accurately assessing their reliability level are essential for maintaining national security and stability and safeguarding people’s property. Because of its features of a high control accuracy, ample output torque and fast response, it has been widely used in the defense industry and high-tech content civil enterprises [2]. However, the direct-drive electro-hydraulic servo system, as the core of the whole electromechanical system, suffers from degradation and reduced reliability, which constitutes a major hazard to the safety performance of the electromechanical system. Therefore, ensuring its service life and operational reliability under various complex working conditions is particularly important.

The direct-drive electro-hydraulic servo valve is an advanced form of electro-hydraulic servo valve development. It avoids the working parts through the direct drive method. The operation of the direct-drive electro-hydraulic servo valve is characterized by the cancellation of the intermediate transmission link, which avoids the hysteresis problem. By relying on the precision of the system drive and the precision of positioning, mechanical wear is reduced and the reliability of the electromechanical system is improved. At present, the world of direct-drive electro-hydraulic servo valves and their spool and sleeve performance, as well as the reliability of the lack of systematic life prediction methodology research, for all types of control valves, is a limited area of study [3]. Only a few scholars have examined the nozzle servo valves for the physical component mechanism degradation of fault detection and diagnosis. Failure indicators are obtained by analyzing the physical wear mechanism of servo valve components such as torque motors, hydraulic amplifiers, and feedback components. However, servo valves have many independent components, each of which have their own unique structure, and the structure of different servo valves is also different. Theoretical derivation alone cannot fully explain the complexity of servo valves in the working environment. In industrial practice, it is also difficult to observe the situation and state of each component as the servo valve working time changes. Therefore, for servo valves, the spool and sleeve often experience relative displacement and friction with the working fluid, corresponding to a shorter life span that is affected by external forces and prone to failure. Therefore, research on the performance degradation and life prediction of servo valves mainly focuses on the valve spool and valve sleeve. Performance refers to the nature and characteristics of the valve. Regardless of the type of servo valve or other control valve, the spool and sleeve have roughly the same properties. They are common performance characteristics are understood through analogy with human lung capacity, muscle strength, etc. Therefore, selecting servo valve performance over operating time as an indicator for judging servo valve degradation will not be limited to specific sizes, structures, and operating conditions. The use of servo valve performance to infer performance degradation and life prediction laws is closer to the actual situation and more applicable to various electromechanical fields and application scenarios.

Although a step-by-step analysis of the physical components can be used to make theoretical derivations regarding the performance degradation and life prediction of servo valves, if it is to be widely used in different fields of work, this method is more time consuming and energy consuming, and the theoretical analyses obtained are more limited and may not necessarily match with the actual situation. Therefore, with the rise of artificial intelligence and machine learning in recent years, the performance parameters obtained from actual experiments of servo valves are analyzed and predicted by combining the theory of neural networks and big data time series. In the current prediction field, Hu Qiguo et al. [4] used the analytical idea of total division; they first carried out the collection empirical analysis, then carried out the chunk splitting, and combined it with the Harris Hawk optimization algorithm to establish a rolling bearing life prediction model, and proved its accuracy through experiments. Zhang Yan et al. [5] from Chongqing University proposed a novel algorithm model using a multi-scale variational particle swarm optimization algorithm to select and optimize parameters, and verified its effectiveness. Xu Yanlei et al. [6] proposed a rolling bearing life prediction model by combining a switching conversion Kalman filter for timed monitoring and data correction, and verified its accuracy. In addition, Yin Aijun et al. [7] from Chongqing University designed a variational autoencoder using probabilistic analysis and variable evaluation, which had high entropy data characteristics and showed good robustness and stability in the life prediction of data-based bearings. Zhou Chen et al. [8] evaluated and predicted the life of the damping capacitor, the main component of the converter valve. They applied multi-frequency parameter pilot assessment and equivalent temperature rise, and considered the operating conditions of the components, especially the operating temperature. By improving it, they proposed a life assessment model on the relationship between temperature and thermal resistance, and verified its validity through experiments. Hao Yan et al. [9] from Beijing Jiaotong University used CFD simulation to describe the wear mechanism of the hydraulic amplifier of the inflow jet valve. The internal wear distribution was obtained according to the Oka model, and the results showed that the wear of the deflector jet valve could be divided into four regular distributions. They also established a relationship between the degree of contamination of the hydraulic fluid and the degree of wear, and proposed a new failure criterion for deflector jet valves for the life analysis of the valves and derived a life prediction formula. Then, scholars Meng Jianwen et al. [10] used empirical mode decomposition combined with particle filters to predict the early lifespan of lithium-ion batteries, and used function standard deviations to verify the feasibility of using filters to predict the lifespan of lithium-ion batteries to some extent. Qiao Zijian et al. [11] also proposed the use of multi-objective optimization with coupled neurons and genetic algorithms for early weak fault identification of mechanical components; experimental verification showed that it had a better recognition performance than filters.

Araujo J A et al. [12] also addressed multi-axis fatigue life prediction in electronic systems, considering size and gradient effects, as well as the influence of micromotion wear, on the life estimation procedure. The results show that multiple axes’ fatigue modeling can estimate mechanical parts’ life under micromotion conditions. Bolotin VV [13] discussed the problem of regularity studies on the reliability of life prediction subjected to irregular or random error stress cases. Using cross-talk theory, he developed a stochastic model explaining the interrelationship between damage accumulation and crack propagation, and performed life prediction. John Andrew Newman [14] developed a life prediction method for stress intensity factors and Walker’s equation for bearing stresses, proposing different crack growth methods. Although the method provided good prediction results on the data for configurations after connection, it was not applicable for the unconnected case. Khorasgani Hamed et al. [15] developed an integrated approach for system lifetime prediction in unknown and stochastic situations. By first proposing an estimation scheme and considering the prediction of future trends, the purpose of lifetime prediction could be converted into a stochastic distribution problem over the system’s life cycle. Finally, they applied data from several cases to compare the error and accuracy of the method. Hongmei Liu et al. [16] proposed a combination of support vector machines and Elman observers for fault monitoring to address the problem of fault diagnosis and prediction of large hydraulic systems. However, this observer could only be applied to highly seasonal periodicity and greater fluctuation of random errors. In vehicle engineering, Xingyan Yao et al. [17] used data acquired from the vehicle’s of individuals connected to the port, through sensor signal processing and conversion; they then connected them to the Labview host computer, which established a set of real-time observations and an early warning system for vehicle shock absorbers. However, this system relied too much on physical experience and real-time recording to predict and analyze future trends.

In summary, it can be understood that the current direct-drive electro-hydraulic servo valves have achieved rapid development in various technical indicators and safety and security, and are widely used in aerospace, military equipment, and other high-tech fields. However, due to the complexity of the structure of direct-drive electro-hydraulic servo valves and the diversity of operating conditions, their durability life and reliability are affected to varying degrees. These effects directly constrain the overall stability and safety of high-tech fields such as aerospace and military equipment, and can even cause direct economic losses and casualties. However, there is currently a lack of systematic life prediction methods for direct-drive electro-hydraulic servo valves and their spool and sleeve performance and reliability. There is a lack of understanding of the changes in the internal performance of servo valves due to wear and tear, as well as the results of these changes, which makes it even more difficult to make use of the servo valves’ performance rules to guide the life of the servo valves or to formulate the failure criterion. For its performance degradation and life prediction aspects, combining the popular neural network in artificial intelligence to construct the prediction model and the prediction analysis of servo valve performance have been considered. The advantage of this type of method lies in the performance of the research object, so once this type of method is applied to the performance of the servo valve, this means that it can be extended for use on other control valves, which will be effective for hydraulic control valves in the aerospace field. Secondly, the use of big data for neural network predictive analysis, because it is based on data obtained from the actual application of servo valves and its predictive effect is closer to the real value and the actual situation than other purely theoretical analyses, can be considered to be directly referenced by the aerospace field. This paper combines the author’s previous research [18,19] to design and improve the performance degradation experiment of servo valves using pressure difference, opening, and oil contamination as the pre-influence conditions. Experimental data are obtained through long-term experiments, and a life prediction model is constructed based on the performance parameter data obtained from the investigation combined with neural networks. The accuracy of the prediction results was quantified and analyzed through error calculation, verifying the feasibility and accuracy of this life prediction model in predicting the service life of direct drive electro-hydraulic servo valves. This provides a theoretical basis for ensuring the safe and stable development of high-tech fields such as aerospace and military equipment.

## 2. Experimental Design and Data Analysis of Performance Degradation of Servo Valves

In previous studies [18,19], it was found that direct-drive electro-hydraulic servo valves are mainly affected by erosive wear and the degree of wear is different because of different openings, differential pressures, and oil contamination. In the first stage of the experiment, pressure gain and leakage were selected as the performance parameters of the servo valve and thus the exponential smoothing and ARIMA model algorithms were chosen for prediction and analysis. However, because the requirements of the Chinese national standard GJB3370-1998 “General Specification for Electro-hydraulic Flow Servo Valves for Aircraft” [20] are known, the standard life of the servo valve must be more than 600 h; the first stage of the experiment was only 500h for time and equipment reasons, so the purpose of this chapter is to lengthen the servo valve operating time to 800 h based on the first stage (aiming to achieve performance parameter data that exceed the standard operating time requirement of 600 h). Moreover, because the performance indexes of servo valves are not less than five categories, the performance parameters of pressure gain and leakage were selected for the first stage, which are the two most commonly used parameters. This experiment was designed to continue to expand the research object and sample size, and was then combined with more advanced neural network algorithms to predict the life of servo valves, which could be better for this life prediction theory. This experiment was designed to realize the life prediction analysis of servo valves through the test data of the other three key indexes of servo valves: overlap, threshold, symmetry, and so on.

### 2.1. Arrangement of Experimental Facilities

**Figure 1:**
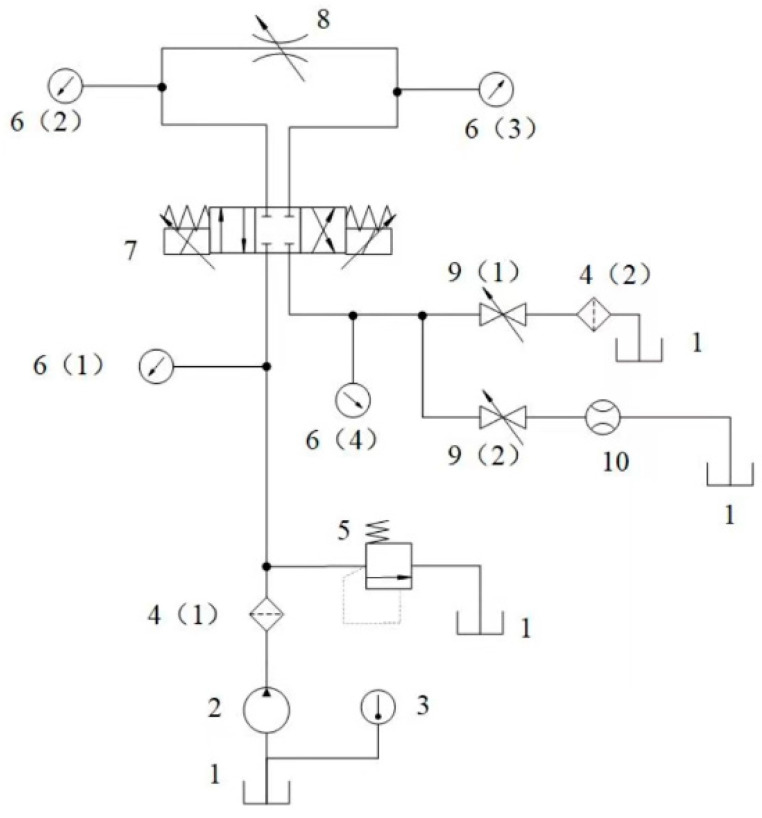
Schematic diagram of the servo valve performance degradation experiment: (1) tank; (2) hydraulic pump; (3) temperature gauge; (4) filter; (5) relief valve; (6) pressure gauge; (7) measured direct-drive electro-hydraulic flow servo valve; (8) throttle valve; (9) stop valve; (10) flowmeter.

Figure 1 shows the schematic diagram of the servo valve spool and sleeve erosion and wear experimental system, as well as the original experiments in the same testing platform and servo valves [19]; solid throttle valves, globe valves, pressure gauges, and other equipment will not be elaborated on. Hydraulic pump 2 is the main pump of the whole flow control system and is responsible for the supply of the working medium or hydraulic oil. Thermometer 3 is used to monitor the initial temperature of the hydraulic oil and can monitor the oil temperature change at any time during the experiment, where two filters (4 (1) and 4 (2)) are used to filter the large diameter solid particles in the oil circuit. The main function of the relief valve (5) is to regulate and maintain the constant pressure of the whole hydraulic system, so its main working object is to supply the source of the oil—hydraulic pump (2). In addition, the four pressure gauges (6 (1)—6 (4)) are used to detect the pressure of the four ports of the servo valve; connected with the pressure sensor, the pressure signal can be transmitted to the computer. The throttle valve (8) is used to regulate the system oil flow according to the experimental condition, and it can also be regarded as a certain load acting on the measured flow servo valve. Finally, (2) the shut-off valve (9) can ensure the measured flow servo valve returns the port to an on and off state control. When (9 (1)) access (9 (2)) is disconnected, it represents the normal working fluid state of the experiment, that is, the measured flow servo valve erosion and wear experiments continue to be carried out. When the measured flow servo valve (7) is in a neutral state, (9 (1)) will be disconnected from (9 (2)), then the flowmeter (10) can be used to detect the zero leakage of the measured flow servo valve, and the flowmeter can be connected to the flow sensor, so that the leakage signal can be input into the computer. In addition, the spool of the servo valve can also be equipped with position sensors for measuring displacement and overlap, etc.

**Figure 2:**
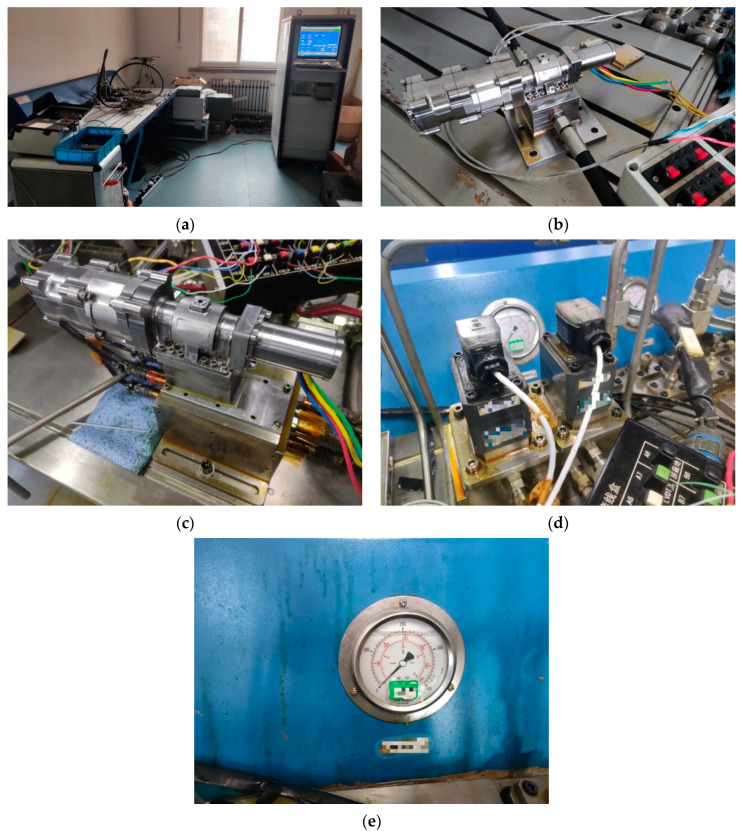
Layout diagram of performance degradation experiment: (**a**) performance degradation test bench; (**b**) servo valves (DDV valves); (**c**) servo valves and pressure sensors; (**d**) flow sensors; (**e**) pressure gauge.

As shown in the A-point diagram in Figure 2, the valve core and valve sleeve wear test bench mainly includes the pump source, tested servo valve, flow meter, throttle valve, globe valve, heat exchanger, pressure gauge, thermometer, and other related pipeline systems; Figure 2b–e shows the physical images of the servo valve (DDV valve) and its valve core and valve sleeve, as well as the physical images of the pressure and flow sensors.

In this test, the performance of each servo valve will be examined considering the effects of erosion and wear, which cause changes in the value behavior. To accomplish this, sensors are employed to capture real-time performance parameters, which are then recorded and processed by a signal computer. The purpose of this experiment is to test and record the performance parameters of direct-drive electro-hydraulic servo valves considering the extension of the working time. We then selected the servo valve performance in the overlap, threshold, and symmetry as our experimental object parameter indicators, so as to obtain the servo valve work of 800 h for performance parameter change data.

### 2.2. Setting of Experimental Conditions, i.e., Working Conditions

**Figure 3:**
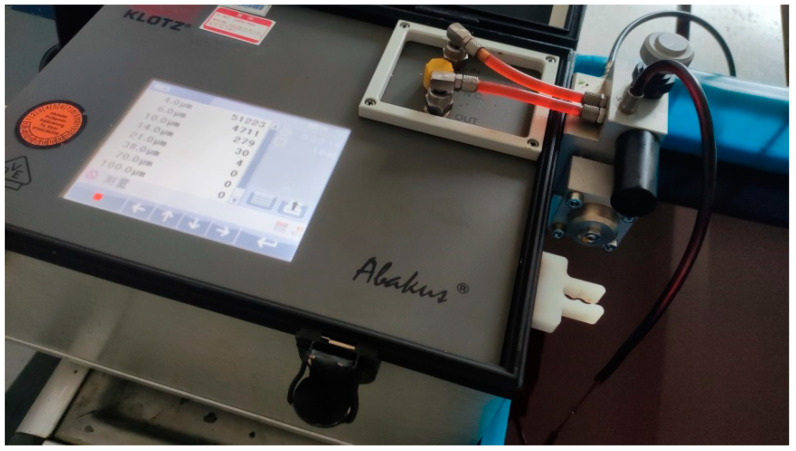
Particle counter.

We used the same setup as a previous study [19], and the oil contamination, opening degree, and differential pressure were still used as the orthogonal setup parameters for the experimental conditions. In the previous study [18], the erosive wear was explored as the main wear of the servo valve spool and sleeve, with opening degree, differential pressure, and contamination degree being the main influencing factors. Therefore, the erosion wear of the servo valve was simplified in this experiment, and the diameter of the maximum number of solid particles in oil was used to reflect the different oil pollution degree. Considering the actual application environment of the DDV valve, four different pollution levels of hydraulic oil were proposed in this experiment: GJB420B-6 grade, GJB420B-7 grade, GJB420B-8 grade, and GJB420B-9 grade. The experiment was conducted using a particle counter to input and record the oil contamination levels, as shown in Figure 3.

In addition, temperature may also affect the degree of erosion and wear of the servo valves. The impact of temperature, especially high temperatures, on the stability and safety of industrial systems is a worthy consideration in the industrial field. For example, in terms of materials, thermal barrier coatings (TBC) are widely used to extend the lifespan of components. Experts [21,22] have found that in high temperature environments of 718 °C, high temperatures lead to an increase in the oxidation cycle of this material layer, affecting the components’ lifespan. However, considering that it is difficult to control the temperature in the experiment, and according to the literature [18], it can also be concluded that temperature has less influence on the erosion and wear of the servo valve compared with other factors, in this study, temperature was not taken into account as one of the experimental working conditions. Instead, the initial temperature of the oil fluid was controlled at around 35 °C, which is a commonly used value in the field of aviation and aerospace. The entire experimental working conditions were set as shown in Table 1. According to GJB3370-1998 “General Specification for Aircraft Electrohydraulic Flow Servo Valves” [20], it is known that the servo valves needed to have a total life of 600 Fh, for which high pollution experiments were carried out, so each sample experiment was carried out for 800 h, and the spool valve sleeve was disassembled for cleaning and decontamination treatment in intervals of 80 h.

### 2.3. Experimental Manipulation

The tested direct drive servo valve (DDV valve) prototype was mounted on the test bench, the inlet and return switches were kept open, the oil pump regulated the inlet pressure, and the control chamber was controlled by a solenoid valve to control the on/off state between control chambers. The automation controller controlled the force motor (FM) movement of the DDV valve, the oil pump, and the solenoid valves.

(1) Performance degradation experiments

The solenoid valve command was turned on to keep the DDV control chamber in communication. The oil pump regulated the oil feed pressure to the specified value. Oil contamination was monitored in real-time by a particle counter.

The DC command was input to the FM through the controller to drive the valve spool to move to the specified position to keep the valve sleeve window at a fixed opening, and to carry out the long-time performance degradation test under various working conditions.

(2) Off-load performance testing

Performance degradation test: Approximately every 50 min, the solenoid valve disconnects and the oil circuit between the control chambers is cut off. The oil pump regulates the inlet pressure to the DDV rated pressure.

The controller inputs a complete cycle of sinusoidal rated command signal to the DDV, causing the spool to travel a full working stroke. The two-control-chamber pressure with the command change curve is recorded, and the two curves show the difference. Taking the difference curve rated pressure as a ±40% section of the calculated slope results in the DDV pressure gain.

The curve of the total flow rate from the inlet to the return port is recorded as a command function to obtain the complete internal leakage cycle curve, and the peak of the curve is used as the DDV internal leakage.

The controller feeds the DDV a full cycle of a 10% rated command sinusoidal signal. A curve of spool displacement versus command is recorded and the difference in command at the widest part of the curve is the DDV threshold.

(3) Open load performance test

At the end of the off-load performance test, the solenoid valve command is turned on and the oil circuit between the control chambers is re-communicated. The controller feeds the DDV a full cycle sinusoidal rated command signal that causes the spool to travel a full working stroke.

The curve that records the change in DDV flow compared to the command is the no-load flow curve. The ratio of command difference to rated command at the widest part of the curve is taken as a percentage, i.e., the DDV hysteresis loop.

The nominal flow gain lines for both polarities are calculated from the no-load flow curves, and the difference between the calculated slopes is taken as a percentage, which is the symmetry of DDV.

At the end of the test, the oil pump adjusts the inlet pressure to the specified value for the test condition. The controller inputs a DC command to the FM to drive the spool to the specified position to continue the performance degradation test.

**Figure 4:**
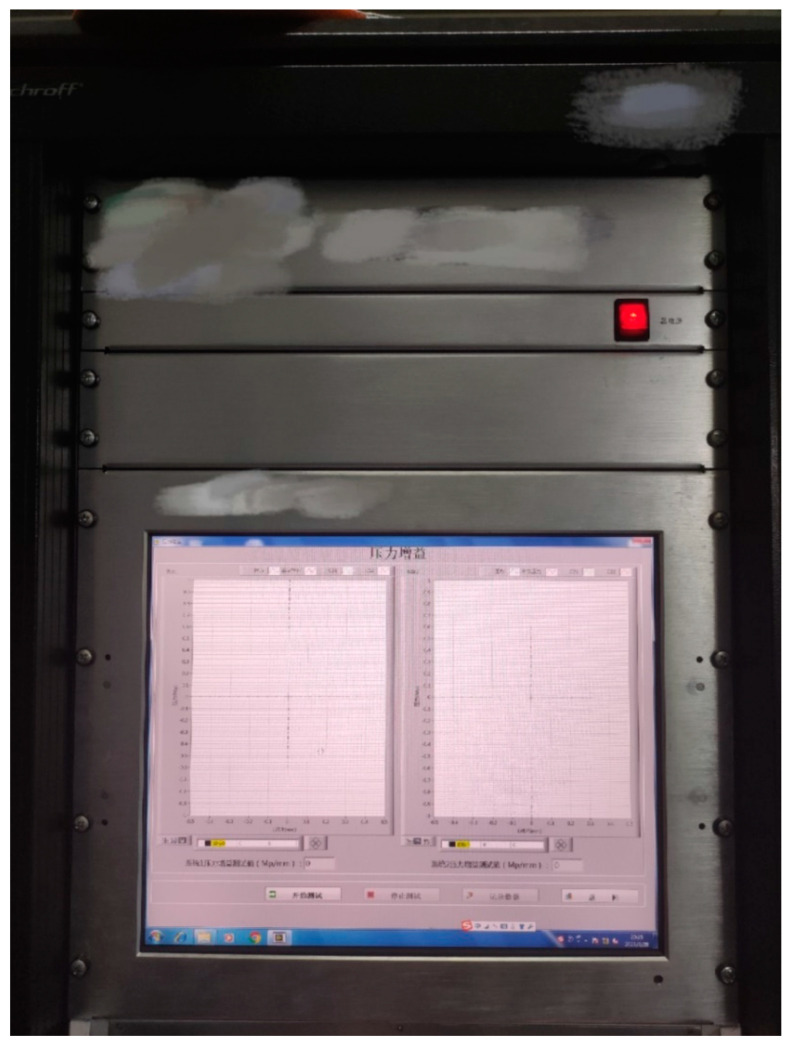
Automated test equipment.

In the experiment, the sampling interval was 80 h for one cycle, and a computer was used to monitor the data in real time, approximately every 50 min, during weekly periods. The prototype of the direct-drive electro-hydraulic servo valve (DDV valve) under test was mounted on the experimental bench, with the inlet and return switches kept open, the inlet pressure regulated by an oil pump, and the on/off control between the control chambers controlled by a solenoid valve. The automated controller controlled the force motor (FM) movement of the DDV valve, the oil pump, and the solenoid valves. The panel of the automated test equipment, shown in Figure 4, is responsible for the input and output of all signals and the performance testing of the DDV valve, such as the overlap, threshold, and symmetry.

### 2.4. Data Analysis

**Figure 5:**
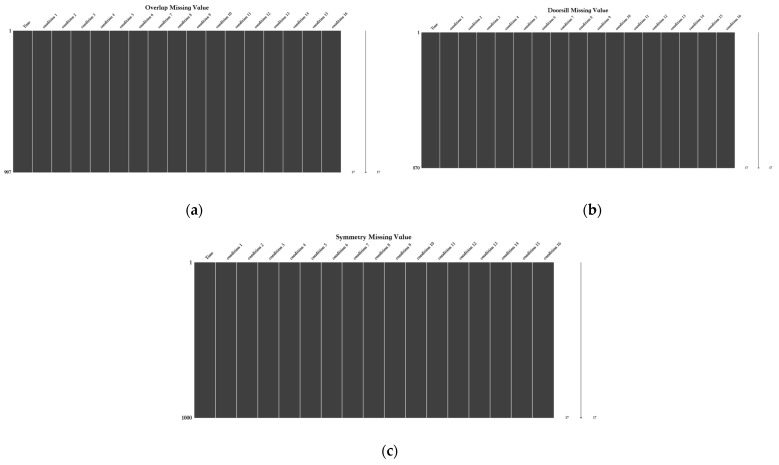
Data information missing map: (**a**) overlapping, (**b**) threshold, and (**c**) symmetry.

The experimental data contain 16 groups of working conditions, i.e., 16 groups of conditions under which the experiments were carried out, and the three dimensions of overlap (mm), threshold (mV), and symmetry (%) of the servo valves were obtained under each group of experimental conditions for 800 h of testing time, respectively. From the data, it is known that the data belong to the time series data, and the first column indicates time. After obtaining the data, the first thing that needs to occur is analyzing the data exploratively, such as the dimensions of the data, and whether the data are missing or not. After analyzing the data, it is known that the amount of data for the three dimensions of data are as follows: overlap (997), threshold (870), and symmetry (1000). Firstly, observe the basic form of the data and carry out the missing value analysis of each data; from Figure 5, it is clearly seen that there are no missing values in the data of the three dimensions.

**Figure 6:**
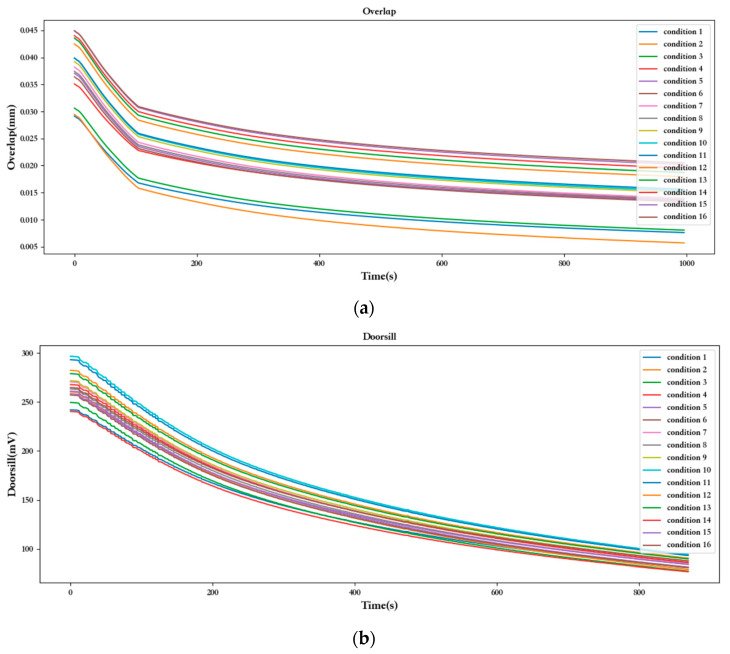

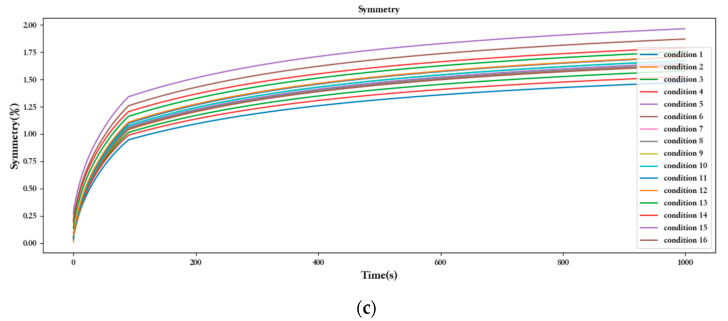
Experimental data trend chart: (**a**) overlap; (**b**) doorstill; (**c**) symmetry.

Further analyses of the trends in the time series of each dimension of data collected through the experiment, starting with the overlapping control valve performance parameter data are as follows:

As shown in Figure 6a, the difference between the overlapped servo valve performance parameters is relatively small, in which the data for conditions 2 and 3 and 11 are low, and the data for the rest of the conditions are relatively small. Overall, with the servo valve work, erosion, and wear, its overlap performance shows a gradual declining trend. When analyzing the reason for this from a physical theory point of view, it may be caused by an increase in gap between the spool valve sleeve and other parts of the servo valve due to wear. However, as the overlap amount is not significant under different operating conditions, it is difficult to conclude which operating conditions influence the overlap most. Of course, it can also be observed that the more contaminated the servo valve, the greater the initial overlap.

Similarly, Figure 6b shows that the difference between the threshold performance parameters of the servo valve data is also very small. With the increase in operating hours, the difference between the threshold of different working conditions was even smaller. The initial threshold value of the servo valve was mainly related to the differential pressure and opening, with a particular emphasis on differential pressure. This can be observed from the data graph, where the differential pressure was larger in several groups of conditions, resulting in higher initial values for the servo valve.

Figure 6c shows the symmetry of the servo valve with the work has an increasing trend. The oil contamination degree has the greatest influence, and the higher amount of pollutant results in the symmetry of the change being more obvious. Thus, the servo valve performance parameters of the change rule resulted in more serious wear, and thus more obvious changes. Therefore, it can be concluded that different conditions in the oil contamination on the servo valve had the greatest impact on wear.

## 3. Lifespan Prediction

### 3.1. Long-Short-Term Memory (LSTM) Neural Network Model

In this paper, we implemented the prediction and analysis of 16 sets of working condition data with different dimensions; considering that the collected data were time-series measurements, we used the machine learning model algorithm of time series, and the common time series algorithms included ARIMA, ARMA, LSTM, GRU, etc. The first two are traditional machine learning algorithms and are based on the linear model, which assumes there is a linear relationship between the time-series data. However, the time series data collected in this paper have the characteristics of non-linearity and insignificant periodicity in the data analysis stage, which may lead to the limited fitting ability of the model. For the latter two data deep learning module algorithms, the GRU model requires greater data support. However, the dataset gathered for this paper comprised less than a dimension of more than 1000; so, this paper used the time series algorithm based on LSTM to establish a prediction model.

LSTM (long-short-term memory), i.e., te long-short-term memory model, is a special RNN designed for the problems of gradient vanishing and gradient bursting that exist in training long sequences. It has been proven by a large number of researches that the LSTM neural network has solved many problems that cannot be solved by the RNN neural network, and has gained further success.

**Figure 7:**
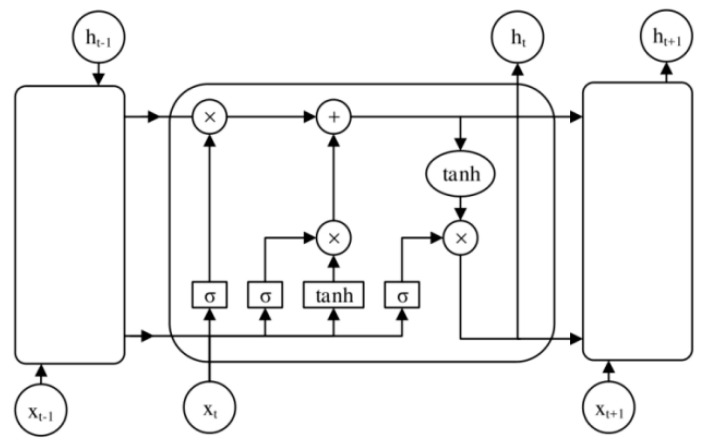
LSTM neural network structure diagram.

The LSTM neural network adds an information storage memory unit that maintains a continuous flow of information so that the gradient does not disappear or explode. Forget Gate, Input Gate, and Output Gate are also constructed to control this memory unit, respectively. These three gates act like filters; the forget gate controls the state information of the memory cell, the input gate updates the state of the memory cell, and the output gate controls the output of the LSTM cell. The LSTM neural network has a structure similar to that of a recurrent neural network, but the difference lies in its working through internal modules. The structure of the LSTM neural network is shown in the following Figure 7:

The LSTM consists of three main gates:

(1) Input gate

The input gate is used to determine new information stored in the cell. The entry gate is used to control the amount of the current network input data image flowing into the storage device, i.e., how much input information is stored in the image. The entry gate consists of two parts, the first part is an “input gate”, which is a signal from 0 to 1 that controls the input to the screen. The second part is a tanh function layer that generates an image of the cell at the current instant, which is added to the cell, depending on the size of the image.

(2) Oblivion Gate

When new information is input, the forgetting gate can now be used if the model needs to forget. The forgetting gate is a key part of the LSTM that effectively suppresses memory preservation and forgetting, and avoids back propagation of the gradient, thus avoiding gradient vanishing and gradient bursting. The forgetting gate determines which information in the state image of the previous cell is deleted by the LSTM. The gate reads the image and the picture, converts it to a number from 0 to 1 using a sigmoid function, and finally this number is multiplied by the state image of the cell to determine what information should be removed from the image. When this number is 1, it means to keep the information of the picture completely and when this number is 0, it means to discard the information of the picture completely.

(3) Output gate

The output values are based on the cell state, but there is a filtering process. Here too, there are two parts of the operation: the first part is an “output gate”, which consists of a sigmoid function, which is between 0 and 1. In the second part, the final output image is generated and multiplied with the control signal image to obtain the final picture of the output value. The output memory controls the influence of the memory picture on the current output picture, i.e., which memory unit will be output at time t. The output value of the memory picture will be calculated by multiplying the output image with the control signal image to obtain the final picture of the output value.

The advantages of LSTM neural networks are mainly in the following three aspects:

① The long-term dependency problem in recurrent neural networks is solved, and it can deal with data sequences with a long time lag. If the information is more important at a certain moment, then its corresponding forgetting gate position will always be kept at a value close to 1. This allows the information at this moment to be passed down the line without being lost, which is one of the reasons LSTM neural networks can handle long sequences.

② It has good convergence properties. The gate of forgetting will control the last memory and merge with the previous memory to form a new memory. The introduction of the “gate” structure allows the LSTM neural network to better store information for a longer time. In this way, the “forgetting” gates can be used to process previous memories, affecting the network output.

③ It is less prone to gradient vanishing or explosion. Recurrent neural networks in the time-based backpropagation in the existence of the multiplier of the activation function derivatives, compared with the LSTM neural network whose corresponding derivatives are not in the form of a product, but by the cumulative way to calculate. This change allows for the gradient vanishing and explosion problems to be solved, while being less likely to fall into a local optimum.

### 3.2. Model Evaluation Methodology

In this paper, the LSTM network model is used for prediction of the measurement data of multiple working conditions in different dimensions, which is a regression problem; the training LSTM model needs to be evaluated to determine their prediction accuracy. In this paper, the following evaluation metrics (MSE, MAPE, and MAE) are used as an assessment of the model performance, and effectiveness:

MSE (mean squared error): the mean squared error is a widely used metric to measure the average prediction error of a model. The smaller the MSE, the smaller the prediction error. The formula is as follows:

(1) $$\mathrm{MSE}=\frac{1}{N}\sum_{i=1}^{N}{\left(y_{i}-y_{i}^{*}\right)}^{2}$$

where *N* denotes the total number of data, $y_{i}$ is the observed value at the *i*th working time point, and $y_{i}^{*}$ is the predicted value at the *i*th working time point.

MAPE (mean absolute percentage error): the mean absolute percentage error is the average of the absolute value of the difference between the predicted value and the actual value divided by the percentage of the actual value. It is calculated by the following formula:

(2) $$\mathrm{MAPE}=\frac{100\%}{N}\sum_{i=1}^{N}\left|\frac{y_{i}^{*}-\bar{y}}{y_{i}}\right|$$

where $\bar{y}$ is the mean value of the pressure gain and leakage samples, $y_{i}$ is the observed value at the first operating time point, and $y_{i}^{*}$ is the predicted value at the first operating time point. The smaller the value of MAPE, the better the predictive ability of the model.

MAE (mean absolute error): the mean absolute error, which represents the average of the absolute errors between the predicted and observed values. MAE is a linear score in which all individual differences are equally weighted on the mean, such that, for example, the absolute error between 10 and 0 is twice as large as that between 5 and 0. It is calculated using the following formula:

(3) $$\mathrm{MAE}=\sum_{i=1}^{N}\frac{\left|y_{i}-y_{i}^{*}\right|}{N}$$

where $\bar{y}$ is the mean value of the pressure gain and leakage samples, $y_{i}$ is the observed value at the first operating time point, and $y_{i}^{*}$ is the predicted value at the first operating time point.

The lower the value of these three metrics, the higher the prediction accuracy and the better the performance of the model.

### 3.3. Data Preprocessing

The LSTM model is very sensitive to the data outline; too large data outline caused by the model training loss cannot decrease and the model does not converge. In the data analysis, we know that the range of the data outline is not the same, especially if the range of values of the threshold is more than 200, but the range of values of the overlapping data is just about 0.03; so, we need to carry out data normalization. Data normalization is the process of scaling the original data, so that its range is limited to a specific range—the common range is [0, 1] or [−1, 1]. The LSTM model has stringent requirements on the data scale, and data normalization can restrict the range of the data to a certain range to avoid the model’s poor performance due to the difference in data scales. In this paper, the Min−Max scaler normalisation method was used. This method is a simple linear normalisation method that scales the data to the range of [0, 1] with the following formula:

(4) $$X^{\prime }=\frac{X-\min\left(x\right)}{\max\left(x\right)-\min\left(x\right)}$$

where *X* is the original data, *X’* is the normalised data, and max(x) and min(x) are the data’s maximum and minimum values, respectively. The normalised data makes the training of the LSTM model smoother.

When performing time series prediction, the dataset needs to be converted to the data format required by LSTM, i.e., the data fed into the LSTM network at each moment is a time series. To achieve this goal, the data need to be converted into a time series using a sliding window approach. Specifically, a time window with a window size of look_back is used as the basic unit, and each moment in the original dataset and the previous look_back moments are formed into a time series as the input to the LSTM network. The data of the next moment of each time series are used as the time series output. In the data analysis stage, it is known that the intergroup differences between overlap, threshold, and symmetry conditions are small, so the look_back parameter is set to 5.

In this paper, we define a function called create_dataset to implement the construction of time series data. The parameter data in the function is the original dataset, and lagTerm indicates the size of the sliding window. The return values of the function are the transformed training and test sets.

Finally, the planned dataset needs to be divided into a training set and a test set so that the model can be trained on the training set and the performance of the model can be evaluated on the test set. The following steps are for the division process: firstly, the size of the dataset is calculated and the first 80% is used as the training set and the last 20% as the test set. Next, partition the dataset into training and test sets and store them in the train_size and test_size variables, respectively. The train_size variable stores the size of the training set which is 80% of the size of the dataset. The test_size variable stores the size of the test set, which is the difference between the size of the dataset and the size of the training set. Next, the original dataset dataset is divided into training set and test set using the slicing method of numpy arrays and they are stored in the train and test variables. In code implementation, the slicing method of numpy array is used to complete the division of the dataset.

### 3.4. Application of the Model

**Figure 8:**
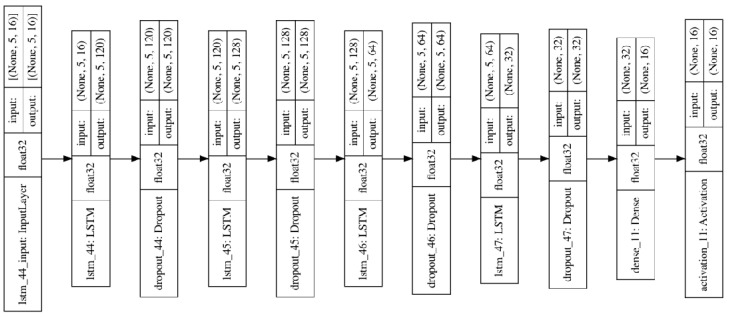
Framework diagram of LSTM model with overlap, threshold, and symmetry.

This paper establishes an LSTM model with overlap, threshold, and symmetry, as shown in Figure 8, using the Keras framework to implement the LSTM model.

This LSTM model contains the following features:

Input layer (for overlap, threshold, and symmetry data): The input to the modeling is the measurement data (look_back) for five time steps, each containing 16 features (16 working conditions).

Three LSTM layers: Each LSTM layer contains a recurrent layer and a forgetting layer. The recurrent layer allows the model to receive and process sequential data, while the forgetting layer allows the model to “remember” critical information and “forget” unimportant information. The first LSTM layer returns a sequence output (return_sequences = True) with 120 neurons, the first LSTM layer returns a sequence output (return_sequences = True) with 128 neurons, and the last LSTM layer returns a single output (return_sequences = False). Sequences = False), and its number of neurons is 64.

Dropout layer: used to reduce overfitting; a dropout layer is added after each LSTM layer with a dropout ratio of 0.2 or 0.3.

Dense layer: the output layer contains 16 neurons for predicting the 16 work measurements at the next time step with 256 neurons.

Activation function: the output layer uses ReLU as the activation function.

Optimizer: Adam, is used to optimise the weights of the model.

Loss Function: Mean squared error.

Indicator: Mean absolute error.

**Figure 9:**
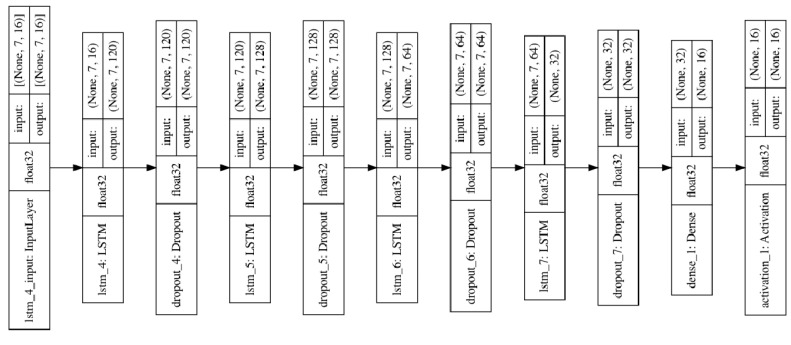
Framework diagram of the LSTM model for pressure gain and leakage.

Based on the experimental data of pressure gain and leakage from the previous study [19], a corresponding LSTM model was established, as shown in Figure 9:

The model in Figure 9 contains roughly the same features as Figure 8, except for different input layers (for pressure gain and leakage data): the input for establishing the model uses measurement data from seven time steps (lookback), with each time step containing 16 features (16 operating conditions).

**Figure 10:**
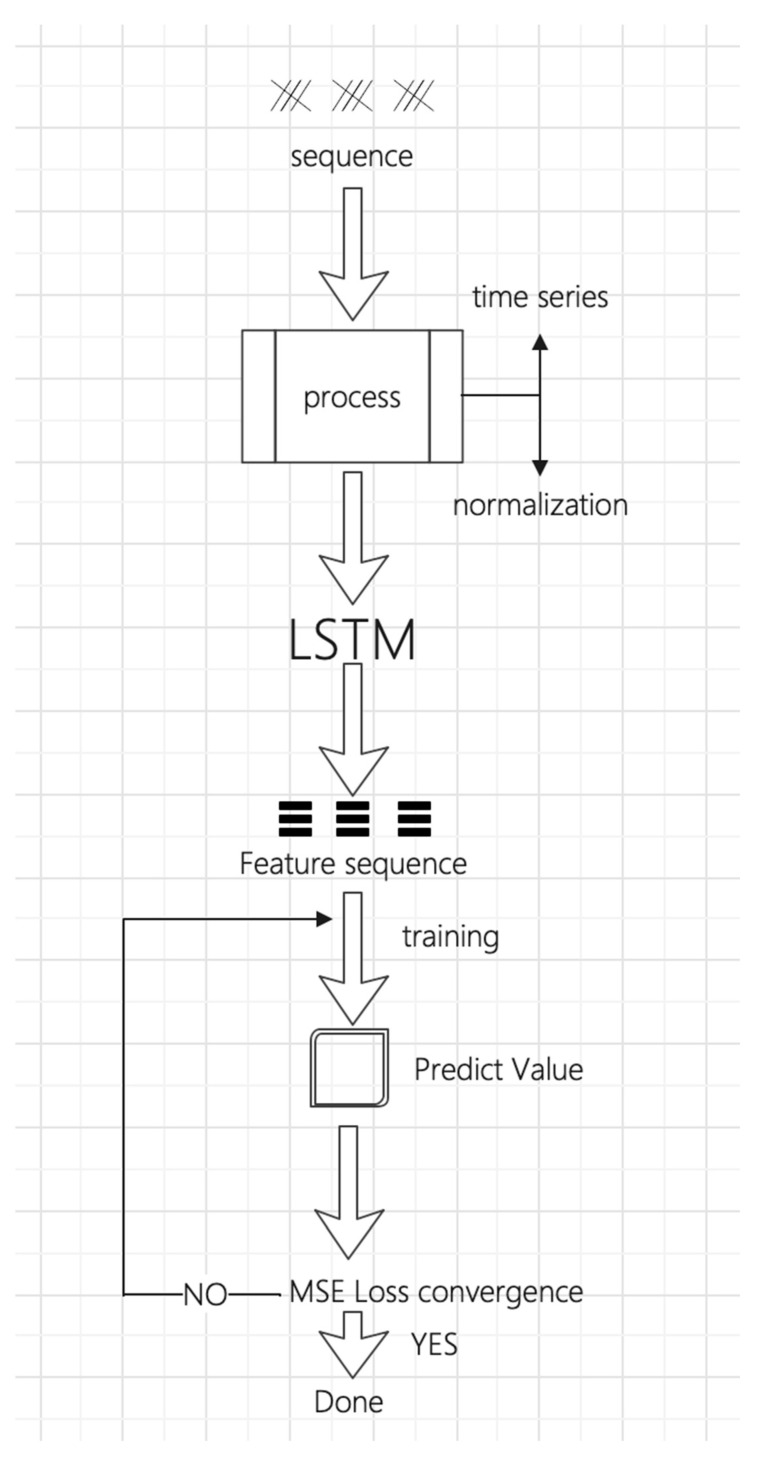
Process framework diagram.

According to refeence [23], the parameter settings of the LSTM model established in this article are shown in Table 2, and the method framework diagram is shown in Figure 10.

### 3.5. Presentation and Evaluation of Training and Prediction Graphs

The fit() function was used to train the model and the training history was stored in the history object, which included the training loss, validation loss, and evaluation metrics. During the training process, callback functions were used to implement early stopping techniques and learning rate decay techniques to improve the generalisation of the model. The parameters were consistent with the established LSTM model, except for the different data, which were aimed at better comparing the prediction effect of the measured data between the working conditions.

In terms of model training, for overlapping, threshold, and symmetry data, due to the minor intergroup differences between data conditions, the established LSTM recurrent neural network models were set with 100 epochs, and the learning rate was set to a smaller 0.001. However, for pressure gain and leakage data, due to the overlap, threshold, and symmetry of the data samples and working hours being smaller, in order to enable the model to learn more information, the number of iterations was set to 400 and the learning rate was set to a smaller 0.000001. The LSTM models with these two different model parameters set an early stop strategy during the training process, which means that if the loss of the model did not decrease continuously 15 times during the training process, the model would stop iterating and retain the model weight at the time of minimum loss during the model training process, that is, the best model would be retained; The batch size was set to 8, which means that the batch size for each training session was 8. At the end of each epoch, the MSE and MAE indicators of the model’s training and validation sets were recorded for an analysis of the model’s performance.

**Figure 11:**
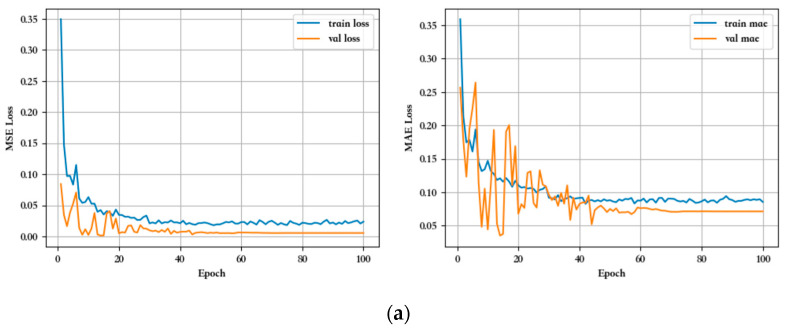

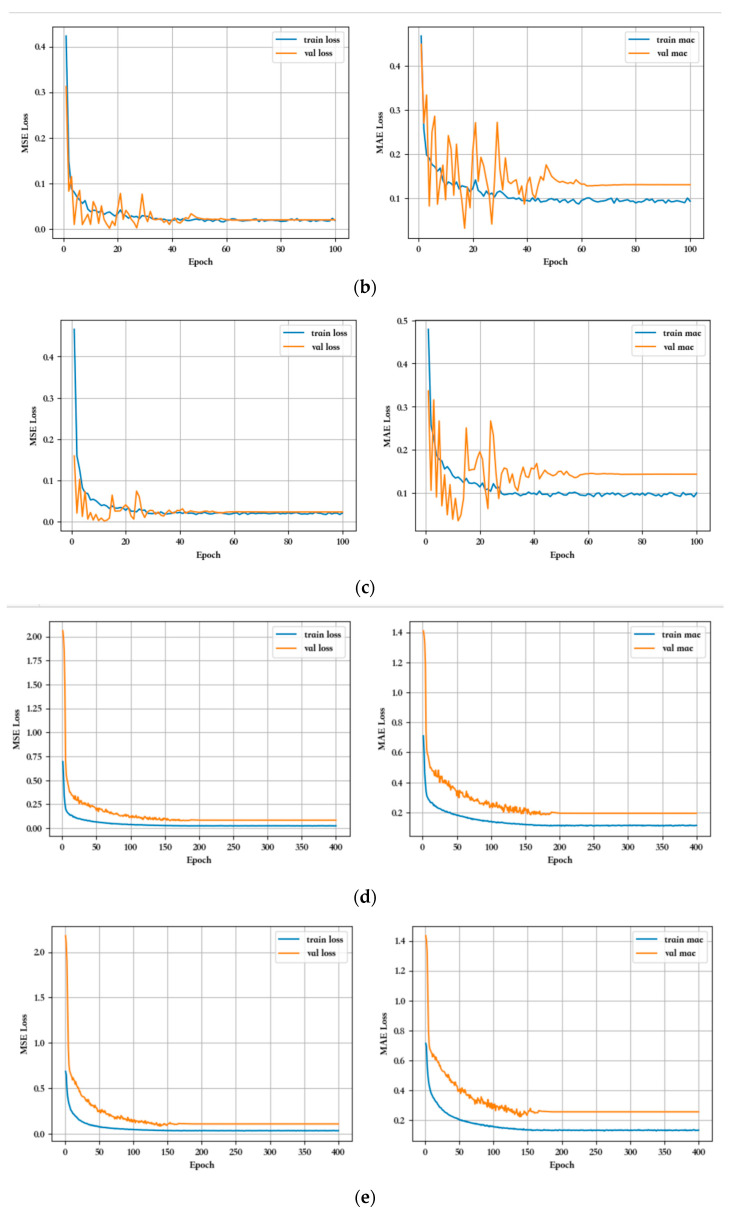
Training iteration log plots: (**a**) overlap; (**b**) thresholds; (**c**) symmetry; (**d**) pressure gain; (**e**) leakage.

Figure 11a–e shows the training iteration log plots for overlap, threshold, and symmetry, respectively.

As shown in Figure 11a, during the 100 iterations, the first 60 or so iterations of the process were unstable due to the fact that the model was still learning the time series information for each of the working conditions in the overlapping dimensions. After 60 iterations, the model region converged, with the MSE loss for training eventually converging to around 0.02 and the MAE metric to around 0.08, and the MSE loss for validation eventually converging to around 0.01 and the MAE metric eventually converging to around 0.07. After 100 iterations, the model became stable, the trained model could be further used to fit the prediction to the divided 20% test data and to calculate various indicators such as MSE, MAE, and several others.

Figure 11b shows the data training for the threshold dimension; what can be seen from the training iteration log plot is that in the process of 100 iterations, similar to the data of the overlap dimension, the first 60 iterations or so were unstable, the model was still in the process of learning, and the learning process in the front was smoother than that of the overlap dimension, which suggests that the data of the individual conditions of the threshold dimension have fewer differences, which is consistent with the conclusions we obtained in the data analysis stage. After 60 iterations, the model region converged, the MSE loss of training finally converged to about 0.01, and the MAE index converged to about 0.1. The MSE loss of validation finally converged to about 0.01 and the MAE index converged to about 0.12. From the training effect, it seems that the training effect of the threshold dimension was slightly worse than the overlapping dimension data, but the model still tended to be stable at the end.

The data training for the symmetry dimension is shown in Figure 11c; what is visible from the training iteration log plot is that during the 100 iterations, similar to the training process for the above data, the first 60 or so iterations of the process were unstable and the model was still in the process of learning. After 60 iterations, the model tended to converge, with the MSE loss for training eventually converging to around 0.03 and the MAE metric converging to around 0.1. The MSE loss for validation eventually converged to around 0.03 and the MAE metric eventually converged to around 0.15. From the training effect, it seems that the training effect of the symmetry dimension was not much different from the above data, and the model also tended to converge at the end.

Because there were fewer data samples and duration pairs for pressure gain and leakage, 400 iterations were conducted to enable the model to learn more time series information. Figure 11d shows that during the training process, the loss of the first 200 iterations gradually converged. In the first 30 iterations, the learning ability of the model was very strong. Finally, after 400 training sessions, the model tended to converge and stabilize. At this point, the MSE loss of the model training finally converged to around 0.01, the MAE index converged to around 0.1, the verified MSE loss finally converged to around 0.015, and the MAE index finally converged to around 0.2. From the perspective of training effectiveness, the training process of the model was ideal, without significant fluctuations.

Similarly, as shown in Figure 11e, the gradual convergence of the first 200 iterations of loss can be seen during the training process. In the first 30 iterations of the model, the data feature information was quickly learned, and finally, after 400 training sessions, the model tended to converge and stabilize. At this time, the MSE loss of the model training finally converged to around 0.01, the MAE index converged to around 0.1, the verified MSE loss finally converged to around 0.015, and the MAE index finally converged to around 0.21. From the perspective of training effectiveness, the overall model training process for leakage was ideal, without significant fluctuations.

**Figure 12:**
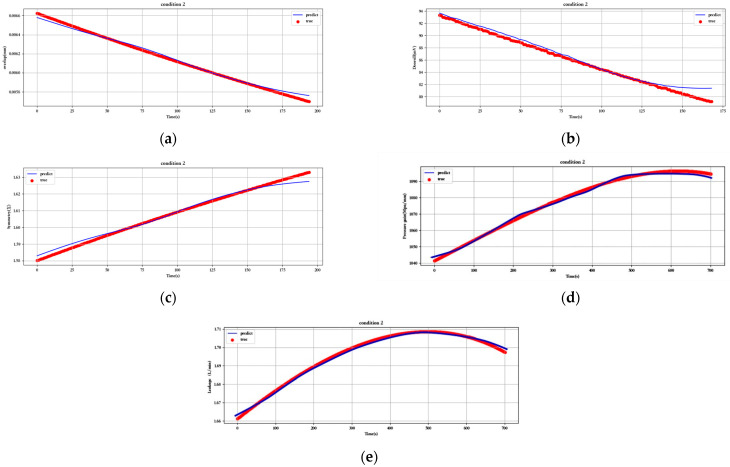
Prediction fitting diagram for overlap, threshold, symmetry, pressure gain, and leakage under condition 2: (**a**) overlap; (**b**) threshold; (**c**) symmetry; (**d**) pressure gain; (**e**) leakage.

**Figure 13:**
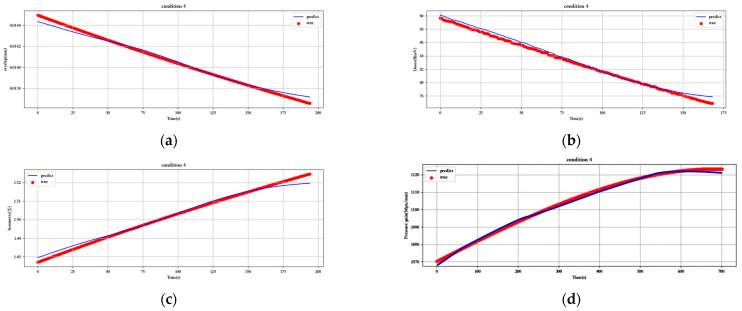

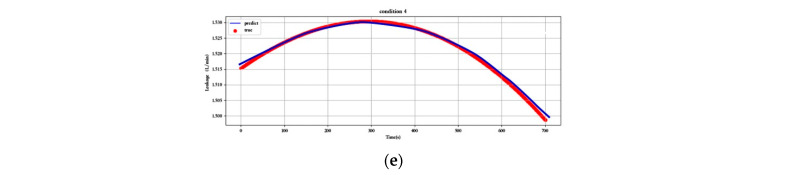
Prediction fitting diagram for overlap, threshold, symmetry, pressure gain, and leakage under condition 4: (**a**) overlap; (**b**) threshold; (**c**) symmetry; (**d**) pressure gain; (**e**) leakage.

**Figure 14:**
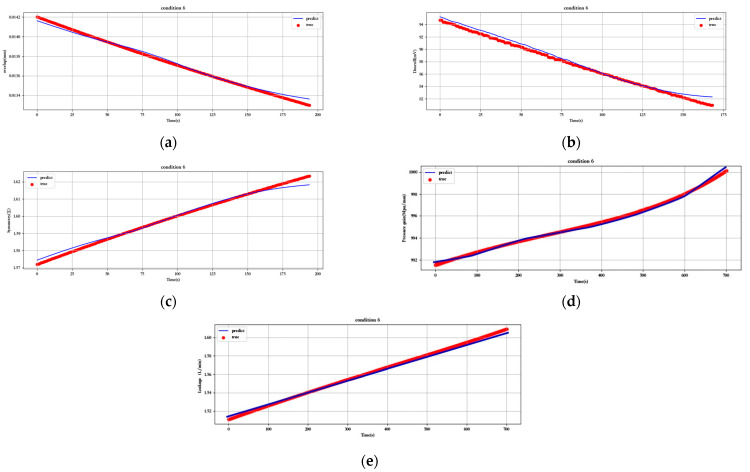
Prediction fitting diagram for overlap, threshold, symmetry, pressure gain, and leakage under condition 6: (**a**) overlap; (**b**) threshold; (**c**) symmetry; (**d**) pressure gain; (**e**) leakage.

**Figure 15:**
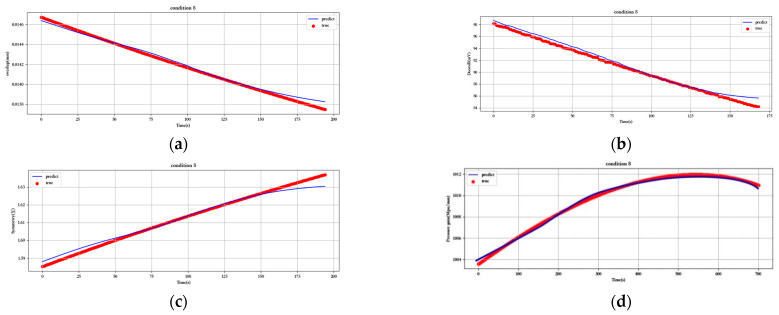

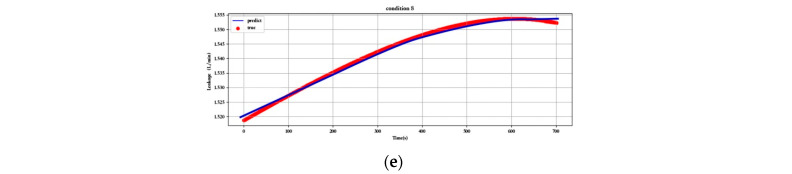
Prediction fitting diagram for overlap, threshold, symmetry, pressure gain, and leakage under condition 8: (**a**) overlap; (**b**) threshold; (**c**) symmetry; (**d**) pressure gain; (**e**) leakage.

**Figure 16:**
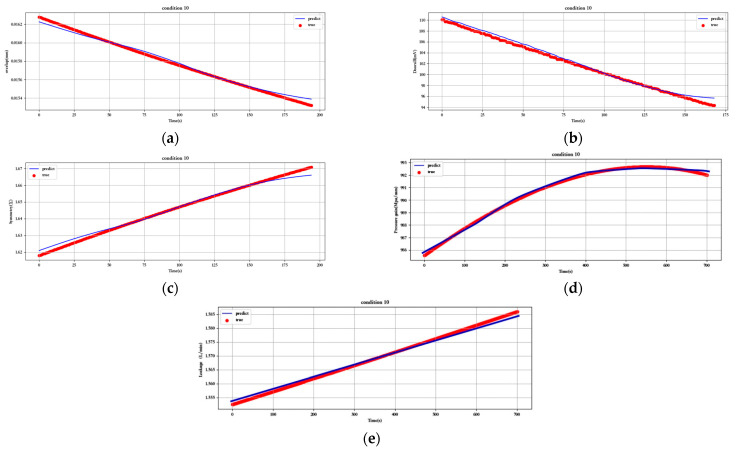
Prediction fitting diagram for overlap, threshold, symmetry, pressure gain, and leakage under condition 10: (**a**) overlap; (**b**) threshold; (**c**) symmetry; (**d**) pressure gain; (**e**) leakage.

**Figure 17:**
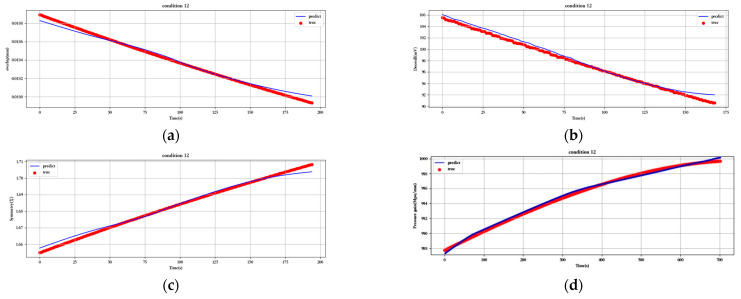

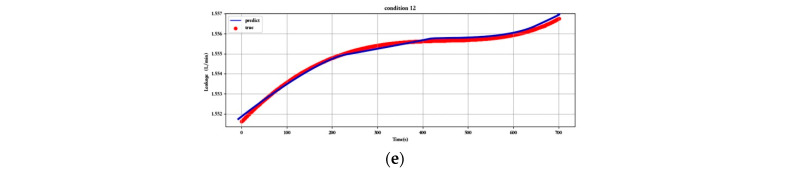
Prediction fitting diagram for overlap, threshold, symmetry, pressure gain, and leakage under condition 12: (**a**) overlap; (**b**) threshold; (**c**) symmetry; (**d**) pressure gain; (**e**) leakage.

**Figure 18:**
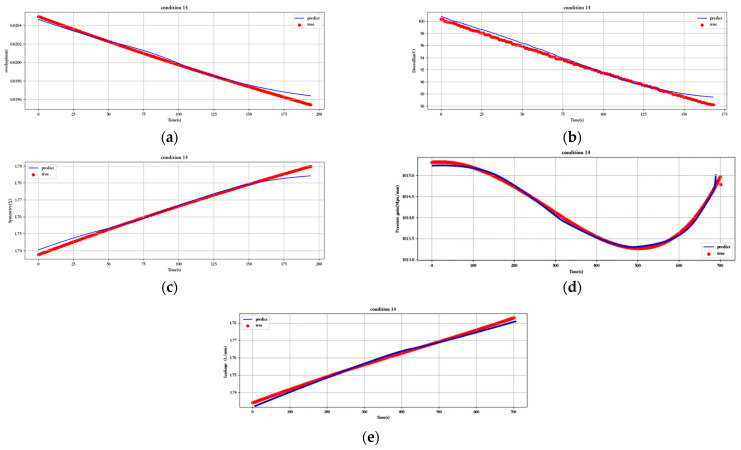
Prediction fitting diagram for overlap, threshold, symmetry, pressure gain, and leakage under condition 14: (**a**) overlap; (**b**) threshold; (**c**) symmetry; (**d**) pressure gain; (**e**) leakage.

**Figure 19:**
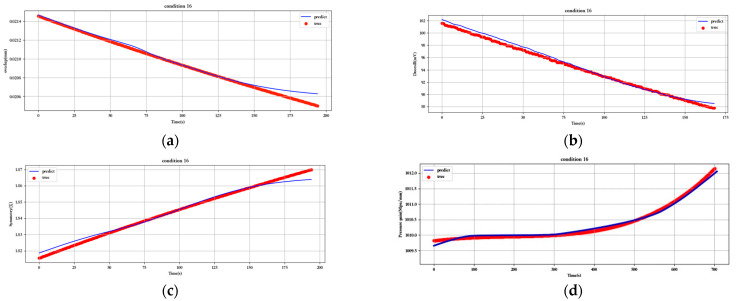

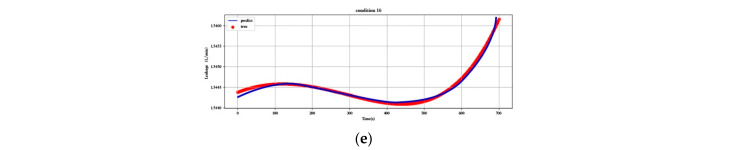
Prediction fitting diagram for overlap, threshold, symmetry, pressure gain, and leakage under condition 16: (**a**) overlap; (**b**) threshold; (**c**) symmetry; (**d**) pressure gain; (**e**) leakage.

Figure 12, Figure 13, Figure 14, Figure 15, Figure 16, Figure 17, Figure 18 and Figure 19 show the predicted fit plots for eight sets of operating conditions, including overlap, threshold, symmetry, pressure gain, and leakage, respectively (because it will take up too much space to display all 16 sets of predicted fit maps for working conditions, we selected the predicted fit maps for groups 2, 4, 6, 8, 10, 12, 14, and 16 as the display). Here, the red scatter is the real value of the test data, the blue curve is the fit of the trained LSTM model to the test data, and its horizontal coordinate represents the time index of the test data; for example, if the test data of the overlap dimension are 187, then the horizontal coordinate represents the 1st test data; the 2nd test data… 187th test data; its vertical coordinate is the specific value of overlap in mm.

Figure 12, Figure 13, Figure 14, Figure 15, Figure 16, Figure 17, Figure 18 and Figure 19 above show the prediction fitting plots of the overlap, threshold, and symmetry for 16 sets of working conditions using the LSTM prediction model. Macroscopically, it can be initially observed from the red prediction curves that the prediction curves are all approximately overlapping with the test set data curves. This indicates that the prediction effect of this model is good considering the overall effect. However, to subdivide it from a micro scale, it relies on the theory of statistics in mathematics to calculate and analyze its error. Through theoretical calculation, the following was obtained by combining all 16 groups of working conditions: in the overlap, MAE was not higher than 2.6 × 10^−^^5^ mm, MSE was not higher than 1.620436 × 10^−^^9^ mm, and MAPE was not higher than 3.844 × 10^−^^3^ mm; in the threshold, MAE was not higher than 5.016 × 10^−^^3^ mV, MSE was not higher than 4.607 × 10^−^^3^ mV, and MAPE was not higher than 5.941 × 10^−^^3^ mV; in symmetry, MAE was not higher than 1.635 × 10^−^^3^ MSE was not higher than 7.20137 × 10^−^^5^, and MAPE was not higher than 8.747 × 10^−^^3^; in the pressure gain, MAE was not higher than 1.76 × 10^−^^4^ MPa/mm, MSE was not higher than 9.15 × 10^−^^4^ MPa/mm, and MAPE was not higher than 1.647 × 10^−^^7^; and in the leakage, MAE was not higher than 5.667 × 10^−^^5^ L/min, MSE was not higher than 9.075 × 10^−^^5^ L/min, and MAPE was not higher than 3.536 × 10^−^^7^.

The experimental data for pressure gain and leakage volume, as well as the error index calculation theory and the previous research literature [19], were the same, while the LSTM prediction algorithm model established in this paper obtained a lower MAE and MAPE than those obtained in the previous literature using the exponential smoothing algorithm model and ARIMA algorithm model, respectively: the MAE for the pressure gain was 2.21 × 10^−^^4^ MPa/mm, MAPE of pressure gain was 1.33 × 10^−^^4^, MAE of leakage was 6.436 × 10^−^^5^ L/min, and MAPE of leakage was 1.58 × 10^−^^4^. It can be seen that the LSTM prediction model was better than the exponential smoothing algorithm and ARIMA algorithm model for predicting servo valves in the aerospace field; this has been shown in previous research literature. This proves that the exponential smoothing algorithm is more accurate than the random forest, support vector machine, decision tree, and other prediction algorithms for this topic. It is proven that the LSTM prediction model developed in this paper is more suitable and accurate than the exponential smoothing algorithm, ARIMA model, random forest, decision tree, support vector machine, and other algorithms.

The above model training and evaluation of the three dimensions of different working conditions found that the prediction of each working condition’s overlap, threshold, and symmetry dimensions fits the data very well. Generally, the fitting prediction effect of each working condition in each dimension was acceptable. To increase the application scenarios of the model, this paper used the trained model to predict the 500 measurements of each working condition in each dimension to anticipate the future and provide early warning. Because of the use of the time series model and the adoption of the single-step prediction method, the reliability of the future prediction effect and credibility decrease over time. This is because long-term predictions need a large amount of forecasting, result in poor prediction results, but can still be useful for a summary of future trends. Using the prediction model for the three dimensions, for the servo valve work of 1500 h, the overlap was not lower than the China Institute of Aeronautics and Astronautics requirements for the threshold of 0.004 mm, the threshold was not lower than 55 mV, the symmetry degree was not higher than 2.5% of the threshold; that is, for the servo valve using the experimental data obtained from the prediction of the service life of at least 1500 h, the value was much greater than the 600-h life requirement of the Chinese national standard GJB 3370-1998 “General Specification for Aircraft Electrohydraulic Flow Servo Valves” [20]. Moreover, the 800 h of experiments directly verified that the life of the servo valve met the Chinese national standard, and the theoretical life of the servo valve was more than 1500 h through the prediction model. Therefore, the LTSM prediction model constructed in this paper would be accepted by the Chinese Institute of Aeronautics and Astronautics, and the prediction accuracy and effect would also meet their requirements.

## 4. Discussion

Through this study, a set of control valve performance parameter data comprising 16 operating conditions is thoroughly analyzed and predicted. These parameters include the three dimensions of overlap, threshold, and symmetry. In terms of predicting and analyzing the data, it is found that the measured data for the overlap, threshold, and symmetry dimensions continue to show a monotonic trend, but gradually stabilize over time. LSTM (Long-Short-Term Memory Network) is used as the predictive model and the data are normalized to cope with the inconsistency of the data measures. By training and evaluating the model, it was found that the overlap, threshold, and symmetry dimensions fit the data very well, with each error calculated to be no higher than 0.2%.

Overall, useful conclusions and insights can be obtained from the prediction of control valve performance parameter data using LSTM models. In practical applications, the prediction results can be used to classify the working conditions and then develop appropriate control strategies. Especially for the data of overlap, threshold, and symmetry dimensions, the prediction model performs very well and can help achieve more accurate control and operation. In addition, a series of effective measures are used to improve the generalization ability and stability of the model during its construction and training. For example, an early stopping technique and a learning rate decay technique are used to avoid the overfitting problem effectively and to ensure that the model maintains better convergence during training. In addition, a sliding window approach was used to convert the data into time series, providing ordered inputs to the LSTM model.

Finally, a prediction model is established based on the data of servo valve performance parameters obtained from different working conditions. Temperature, as a factor affecting the erosion and wear of servo valves, possesses challenges due to the extreme variations in aerospace environments, ranging from low to high temperatures. The intricate task of controlling the temperature led to its exclusion from the study. However, in other fields or occasions (such as [21,22]) the effect of high temperature conditions is also a part of the system stability and the life prediction cannot be ignored.

## 5. Conclusions

This study provides an important reference for the operation and maintenance of control valves through an in-depth analysis and prediction of the control valve performance parameter data. The ability to accurately predict the measured data for each operating condition in three dimensions is obtained through the training and evaluation of the LSTM model. In the dimensions of overlap, threshold, symmetry, pressure gain, and leakage, which are the five servo valve performance parameters, the established prediction model shows excellent fitting results and predicts the future measurement data accurately. In practical applications, this will help operators better grasp the state of control valves, adjust control strategies in a timely manner, and improve the operational efficiency of equipment.

Specifically, from the experimental results, it can be seen that as the working time progresses, the degree of erosion and wear on the valve core and valve sleeve of the servo valve increases. Overall, it is observed that the performance parameters of the servo valve show a slow trend of change under different working conditions, and the rate of change is generally higher under high pollution (level 9) conditions than under other conditions. Through the calculation and analysis, the predicted values of various performance parameters of the servo valve by the prediction model are compared with the experimental test set data, and the error of each item is less than 0.2%. The model meets industrial needs by comparing the two dimensions of accuracy and prediction trend.

This paper synthesizes the performance parameters of servo valves for design experiment validation and prediction, which improves the reference and guidance for the performance analysis of servo valves and provides a reference for the development of use and failure standards for direct-drive electro-hydraulic servo valves in aerospace and other industrial fields. Of course, for the prediction of a longer period in the future, the prediction results may not be accurate enough due to the complexity of the time series information, and thus need to be treated with caution.

In summary, this study has achieved certain results in the prediction research of control valve performance parameter data, primarily through the long time servo valve performance degradation experiments, and the prediction of machine learning algorithms based on big data, discussing the practicality and accuracy of different algorithms in this application, determining and verifying the superiority of LSTM neural network in this time series data, and providing useful information for the operation and maintenance of control valves. Future research can further optimize the parameters and structure of the model and explore more effective prediction methods to improve the accuracy and stability of prediction. At the same time, knowledge from other related fields can be combined to expand the application scope of the model further to provide more comprehensive support for the operation and maintenance of industrial equipment.

## Figures and Tables

**Table 1:** Table of orthogonal experimental conditions.

Serial Number	Oil Contamination Level	Openness/mm	Differential Pressure/MPa	Number of Samples	Number of Measurements	Measurement Interval/h
1	GJB420B-6	0.1	14	1	10	80
2	GJB420B-6	0.2	16	1	10	80
3	GJB420B-6	0.3	18	1	10	80
4	GJB420B-6	0.4	20	1	10	80
5	GJB420B-7	0.1	16	1	10	80
6	GJB420B-7	0.2	14	1	10	80
7	GJB420B-7	0.3	18	1	10	80
8	GJB420B-7	0.4	20	1	10	80
9	GJB420B-8	0.1	18	1	10	80
10	GJB420B-8	0.2	20	1	10	80
11	GJB420B-8	0.3	14	1	10	80
12	GJB420B-8	0.4	16	1	10	80
13	GJB420B-9	0.1	20	1	10	80
14	GJB420B-9	0.2	18	1	10	80
15	GJB420B-9	0.3	16	1	10	80
16	GJB420B-9	0.4	14	1	10	80

**Table 2:** Parameter selection table for LSTM model.

	Type	Input Neurons	Output Neurons	If Dropout
Layer1	LSTM Layer	16	120	—
Layer2	LSTM Layer	120	128	—
Layer3	LSTM Layer	128	64	—
Layer4	LSTM Layer	64	32	0.3
Layer5	Dense Layer	32	16	0.2
Activation:Linear				
Optimizer: Adam, Learning Rate: 0.0001				

## Data Availability

Data sharing is not applicable.
